# Development of an HPLC-based guanosine monophosphate kinase assay and application to *Plasmodium vivax* guanylate kinase

**DOI:** 10.1016/j.ab.2019.03.022

**Published:** 2019-06-15

**Authors:** Liliana Pedro, Megan Cross, Andreas Hofmann, Tin Mak, Ronald J. Quinn

**Affiliations:** Griffith Institute for Drug Discovery, Griffith University, Brisbane, Queensland, Australia

**Keywords:** Guanosine monophosphate kinase, Guanylate kinase, Nucleotide, HPLC, Substrate inhibition, Sigmoidal kinetics

## Abstract

The development of a high-performance liquid chromatography (HPLC)-based method, for guanosine monophosphate kinase activity assays, is presented. The method uses the intrinsic UV absorption (at 260 nm) of substrates and products of the enzymatic reaction (GMP, ATP, ADP and GDP) to unambiguously determine percent conversion of substrate into product. It uses a commercially available C18 column which can separate reaction samples by elution under isocratic conditions in 12 min per run. The kinetics of the forward reaction catalyzed by *Plasmodium vivax* guanylate kinase (*Pv*GK), a potential drug target against malaria, was determined. The relative concentrations of the two substrates (GMP and ATP) have a distinct effect on reaction velocity. Kinetic analyses showed the *Pv*GK-catalyzed reaction to be associated with atypical kinetics, where substrate inhibition kinetics and non-Michaelis-Menten (sigmoidal) kinetics were found with respect to GMP and ATP, respectively. Additionally, the method was used in inhibition assays to screen twenty fragment-like compounds. The assays were robust and reproducible, with a signal window of 3.8 and a Z’ factor of 0.6. For the best inhibitor, an IC_50_ curve was generated.

## Highlights

• Simple HPLC separation of nucleotides involved in the guanylate kinase reaction.
• Direct and unambiguous determination of percent conversion of substrate into product.
• Successful application to *Plasmodium vivax* guanylate kinase (*Pv*GK) activity studies.
• Reaction catalyzed by *Pv*GK found to be associated with atypical kinetics.
• Robust and reproducible inhibition assay for compound screening.

## Graphical abstract

**Figure:**
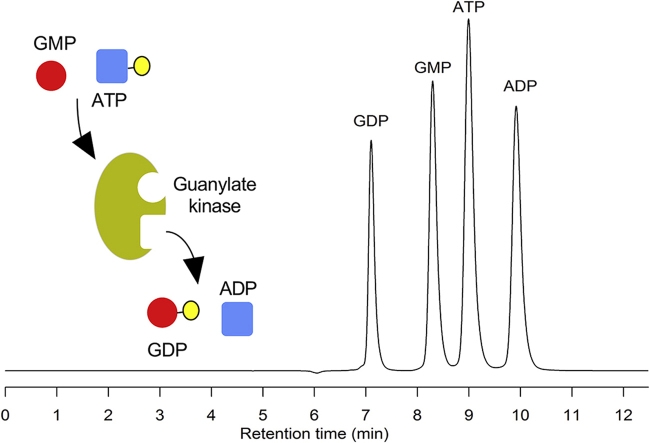

## 1 Introduction

Guanosine monophosphate kinase, or guanylate kinase (EC 2.7.4.8), catalyzes the ATP-dependent reversible phosphorylation of GMP/dGMP into GDP/dGDP, the substrate for GTP/dGTP synthesis. GTP/dGTP, in turn, can be used for nucleic acid or cyclic GMP (cGMP) synthesis. Due to its role in supplying DNA and RNA precursors, guanylate kinase constitutes a potential antimicrobial drug target [1,2]. In many Gram-positive bacteria, its inhibition by guanosine tetraphosphate/pentaphosphate, known as (p)ppGpp, is involved in stress responses [3]. In mammals, guanosine monophosphate kinase is important for the activation of some guanosine-analogue prodrugs (such as acyclovir, ganciclovir, 6-thioguanine and 6-mercaptopurine) used to treat certain cancers and viral infections [4,5].

Recent improvement in our understanding of the biochemistry of malaria parasites has identified many potential new drug targets [[6], [7], [8]]. For antimalarial drug discovery, these are generally proteins that play an important role in parasite survival, are druggable and are absent in humans or, at least, display structural differences that can be exploited in the development of selective drugs [9]. *Plasmodium vivax* (*P. vivax*) is the malaria parasite with the widest geographical distribution and is responsible for about half the cases of malaria outside Africa [10]. It has a dormant hypnozoite stage in liver cells that can cause multiple relapses after a primary infection. For treating the liver stage, primaquine and tafenoquine are currently the only drugs available. Primaquine requires a regimen of at least 14 days, is contraindicated in patients with severe forms of glucose-6-phosphate dehydrogenase (G6PD) deficiency and cannot be given to pregnant women or children under 6 months of age [10]. According to the World Health Organization (WHO), *P. vivax* represents a major challenge to achieve the targets set by the Global Technical Strategy for Malaria 2016–2030, which aims at eliminating malaria from at least 35 countries and reducing case incidence and mortality rates by 90% globally [11]. *P. vivax* guanylate kinase (*Pv*GK) is overexpressed during the liver stage of the *Plasmodium* life cycle, which suggests an important role during this stage and a potential to serve as a good drug target [[12], [13], [14]]. Based on comparison with the BLASTP (protein-protein Basic Local Alignment Search Tool) algorithm, *Pv*GK and *Homo sapiens* guanylate kinase share 41% sequence identity and considerable variation among amino acids that surround the active site. In a high-throughput screening (HTS) binding assay against *Pv*GK, Crowther et al. identified 17 hits (hit rate 0.3%) [13]. Of these, 3 were found to be specific for *Pv*GK, but none of them had inhibitor properties that warranted any medicinal chemistry efforts [13].

Guanylate kinase activity has traditionally been measured spectrophotometrically using a coupled-enzyme system (pyruvate kinase and lactate dehydrogenase in the presence of phosphoenolpyruvate and NADH) [15]. Alternatively, the generic kinase assay of bioluminescent detection of ATP/ADP using firefly luciferase could be employed. The main problem with these and other coupled-enzyme systems is that one needs to ensure that they are not rate-limiting and the observed kinetics are solely due to the target reaction, i.e. are not biased by the detection system [16]. A similar problem exists with other indirect detection technologies (e.g., antibody-based assays), where the detection reagents/components cannot be completely consumed or saturated during the enzymatic reaction [16]. All substrates and products of the guanylate kinase reaction (GMP, ATP, ADP, GDP) are spectroscopically active, absorbing UV light at 260 nm. Therefore, if they can be successfully separated from each other using liquid chromatography, they can be directly detected and quantified. Here, we report the development of an HPLC-based method specific for guanylate kinase activity, which directly measures the concentrations of substrates and products of the enzymatic reaction through their intrinsic UV absorption. Specifically, we demonstrate its application to the study of *Pv*GK enzyme kinetics and to the setup of an inhibition assay for compound screening and IC_50_ determination.

## 2 Materials and methods

### 2.1 Chemicals and reagents

All chemicals were purchased from Sigma-Aldrich (Castle Hill, NSW, Australia) and used as received unless otherwise stated. 96-well V-bottom polypropylene assay plates (AB-0800) were obtained from Thermofisher Scientific Australia Pty Ltd. (Scoresby, VIC, Australia).

### 2.2 Expression and purification of recombinant PvGK

*Escherichia coli* (*E. coli*) Rosetta Oxford strain [BL21*(DE3)-R3-pRARE2] cells transformed with the BG1861 (a pET14b derivative) vector expressing full-length *Pv*GK with an N-terminal hexa-histidine tag were kindly provided by Wesley C. Van Voorhis and Gregory J. Crowther, from the University of Washington. As previously described [17,18], 200 μL of the glycerol stock of transformed *E. coli* cells was inoculated in 200 mL of Luria-Bertani plus (LB+) medium containing antibiotics (100 μg/mL ampicillin and 68 μg/mL chloramphenicol). This culture was incubated overnight at 37 °C under shaking at 180 rpm and then used to inoculate 8 L of LB + medium, supplemented with antibiotics (as above). An in-house adaptation of auto-induction protocol described by Studier [19,20] was used to initiate *Pv*GK expression: 320 mL each of auto-induction mixtures 1 (625 mM Na_2_HPO_4_, 625 mM KH_2_PO_4_, 1.25 M (NH_4_)_2_SO_4_, 125 mM NaCl) and 2 (1.35 mM glycerol, 70 mM glucose, 140 mM α-lactose monohydrate) was added to the culture, which was incubated at 37 °C and 180 rpm until OD_600_ = ~0.6. At this time, the temperature of the incubator was decreased to 15 °C for approximately 48 h. To harvest cells, the culture was subjected to centrifugation at 1000*g* for 30 min at 4 °C. The cell pellet was re-suspended in 200 mL D1 buffer (100 mM NaCl, 1 mM EDTA, 20 mM TRIS pH 8, 0.1% Triton X-100, 1 mM phenylmethylsulfonyl fluoride (PMSF), 5 mM benzamidinium chloride) and stored at −20 °C [20].

For cell lysis, the frozen cells were subjected to three freeze-thaw cycles followed by two cycles of sonication on ice for 4 min using 80% pulsing power and 40% pulsing frequency. The lysate was cleared by centrifugation for 1 h at 4 °C and 100 000 *g*. The supernatant (~350 mL) was loaded onto a Ni^2+^-nitrilotriacetic acid (NTA) resin column (Qiagen, Doncaster, VIC, Australia) pre-equilibrated with wash buffer (20 mM TRIS pH 7, 500 mM NaCl). The flow-through was collected and any unbound proteins were removed with wash buffer (~10 column volumes). The His-tagged *Pv*GK protein (and any other Ni^2+^-binding proteins) was then eluted with elution buffers 1–5 (50 mL of 20 mM TRIS pH 7, 500 mM NaCl with 20, 50, 100, 250 or 500 mM imidazole for buffers 1 to 5, respectively) and collected in five 10 mL fractions per buffer. To locate the protein, fractions 2 and 5 of each elution buffer were analyzed by sodium dodecyl sulfate polyacrylamide gel electrophoresis (SDS-PAGE), which was run at 140 V and stained with Coomassie Brilliant Blue. Fractions containing *Pv*GK were combined and dialyzed against 20 mM TRIS pH 7, 1 mM dithiothreitol (DTT), 5% glycerol. Purification of *Pv*GK was completed by anion exchange chromatography with Q-Sepharose (GE Health, Rydalmere, NSW, Australia). Following dialysis, the protein was loaded onto the pre-equilibrated column (wash buffer of 20 mM TRIS pH 7, 1 mM DTT, 5% glycerol), and the flow-through was collected. After a column wash with 10 column volumes, the column was eluted using a linear gradient of 0–500 mM NaCl in 20 mM TRIS pH 7, 1 mM DTT, 5% glycerol. Fractions of 10 mL were collected and odd-numbered fractions, along with the flow-through, were analyzed by SDS-PAGE. The protein was not retained in the column and was obtained in the flow-through fractions. These fractions were pooled and MgCl_2_ and KCl were added to final concentrations of 2 mM and 50 mM, respectively. The protein concentration was determined by intrinsic absorbance at 280 nm (using an extinction coefficient of 14900 M^−1^ cm^−1^) and an aliquot was concentrated 5 and 10 times with a 10 kDa molecular weight cutoff Amicon membrane (Merck Millipore, Bayswater, VIC, Australia) and re-analyzed on SDS-PAGE (12%) to confirm purity. Finally, the protein was aliquoted in 10 mL tubes and stored at −80 °C until needed.

### 2.3 Instrumentation and chromatographic conditions

HPLC analyses were performed on an Agilent 1100 series apparatus, equipped with a binary pump (G1312A), a micro vacuum degasser (G1379A), a well-plate autosampler (G1367A), an autosampler thermostat (G1330B), a thermostatted column compartment (G1316A) and a variable wavelength detector (G1314A). Chromatographic separations were carried out on a Discovery C18 5 μm, 250 × 4.6 mm column (Sigma-Aldrich, Castle Hill, NSW, Australia), with a flow rate of 0.5 mL/min. The mobile phase consisted of A: 150 mM phosphate buffer, pH 6 and B: methanol (MeOH). The injection volume was 10 μL and the system was run isocratically at 3% B for 12 min. The autosampler was maintained at 4 °C with the column compartment at 25 °C. The detection wavelength was 260 nm.

### 2.4 *Pv*GK enzyme assay

*Pv*GK was assayed at 25 ng/mL (1.06 nM) in 50 mM TRIS pH 7, with 50 mM KCl, 2 mM MgCl_2_ and 0.1 mg/mL bovine serum albumin (BSA). The enzymatic reactions were undertaken in 50 μL volumes at room temperature and quenched with 10 μL 0.1% (v/v) HCl.

To study the enzyme kinetics, time course reactions were performed for the GMP saturation curve, with final GMP assay concentrations of 1, 5, 10, 20, 50, 100, 150 and 250 μM and an ATP concentration of 500 μM. For the ATP saturation curve, reaction time courses were performed with final ATP concentrations of 1, 5, 10, 20, 50, 100, 150, 250 and 500 μM and GMP concentrations of 100 μM. All reactions were conducted in triplicate. Peak areas (*A*) were integrated using the Agilent ChemStation software, and percent conversion was calculated according to the following equation: *%Conversion = A*_*product*_*/(A*_*product*_*+A*_*substrate*_*) × 100*. Incubation times were adjusted to obtain enough data points during the linear phase of the progress curves (%Conversion *vs.* time plot) and to appropriately estimate initial velocities. Initial velocities were obtained as the slope of the linear curve fitted to each progress curve and used to build the saturation curves (plots of initial velocity *vs.* concentration of substrate).

The inhibition assays were performed at room temperature for 20 min. Fragment stock solutions were prepared in dimethyl sulfoxide (DMSO) at 250, 125 or 5 mM and assayed at 5 mM, 2.5 mM or 100 μM final concentrations, respectively. Final DMSO percentage in the enzymatic assay was 2% (v/v). Percent inhibition was calculated using the following equation: *%Inhibition = (%Conversion*_*w/o inhibitor*_ *- %Conversion*_*w/inhibitor*_*)/(%Conversion*_*w/o inhibitor*_*) × 100*. These assays were performed in duplicate and repeated one or two times. All were accompanied by 0% and 100% inhibition controls, the latter obtained by adding 10 μL HCl 0.1% (v/v) before the addition of ATP for reaction start to each well. To evaluate the assay performance and reproducibility over the course of the inhibition experiments, the following formulas were used to calculate signal window and Z′ factor, respectively: *Signal Window = (Mean*_*0% inhibition*_ *- Mean*_*100% inhibition*_ *- 3 × (SD*_*0% inhibition*_*+SD*_*100% inhibition*_*))/(SD*_*0% inhibition*_*), Z’ Factor = 1 - 3 × (SD*_*0% inhibition*_*+SD*_*100% inhibition*_*)/(Mean*_*0% inhibition*_*+Mean*_*100% inhibition*_*)*, where SD is the standard deviation. For half maximal inhibitory concentration (IC_50_) curve, percent enzymatic activity was plotted against the logarithm of the concentration of the inhibitor and fit to the following equation using GraphPad Prism: *%Activity = 100/(1 + 10* ^*(LogIC50 – [I]) × H*^*),* where *[I]* is the inhibitor concentration and *H* the Hill Slope.

## 3 Results and discussion

### 3.1 HPLC method development for assaying guanylate kinase activity

**Fig. 1:**
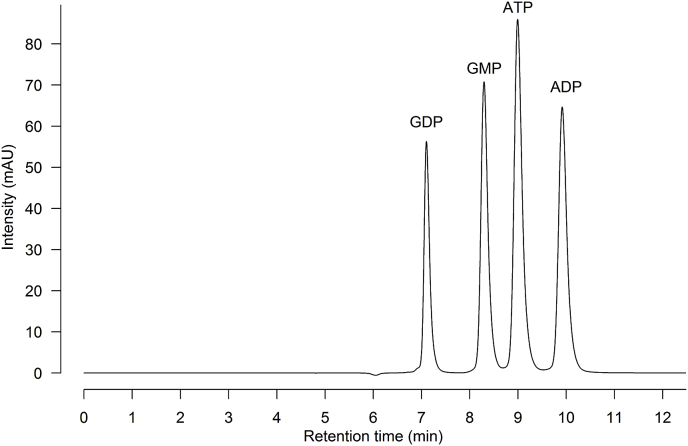
**HPLC chromatogram of GMP, GDP, ATP and ADP nucleotide standard mixture** (50 μM) separated by a Discovery C18 column (5 μm, 25 × 4.6 mm) using phosphate buffer (150 mM pH 6)/MeOH (97:3; v/v).

The ideal assay would directly detect the formation of GDP and ADP catalyzed by the guanylate kinase enzyme, as well as the consumption of ATP and GMP. Even though nucleotides are hydrophilic and challenging to separate on a conventional reversed-phase column due to their poor retention, direct detection of substrates and products would obviate the need of a coupled-enzyme system. Moreover, measurement of product formation is advantageous compared to detection of substrate depletion alone, since the latter is usually not as sensitive and often requires high substrate turnover to obtain acceptable signal/background ratios [16]. To separate GMP, GDP, ATP and ADP by reversed-phase HPLC, the simplest possible method was desirable - that is, one that would not require an ion-pairing reagent in the mobile phase and that could separate samples by isocratic elution. Based on the work of Studzińska and Buszewski [21], several commercially available reversed phase C18 columns, including Prodigy ODS-3 (Phenomenex), Hypersil Gold (Thermo Fisher Scientific), Hypersil BDS (Thermo Fisher Scientific) and Discovery (Supelco), were investigated. Of these, the Discovery C18 column (5 μm, 25 × 4.6 mm) provided the best results. For the mobile phase, both ammonium acetate and phosphate buffer (KH_2_PO_4_/K_2_HPO_4_), run isocratically with 3–5% methanol (v/v), were tested [21]; we found that phosphate buffer (150 mM pH 6)/MeOH (97:3; v/v) provided better separation and peak resolution. Under these conditions, GDP eluted at 7.1 min, GMP at 8.3 min, ATP at 9.0 min and ADP at 9.9 min (Fig. 1).

**Fig. 2:**
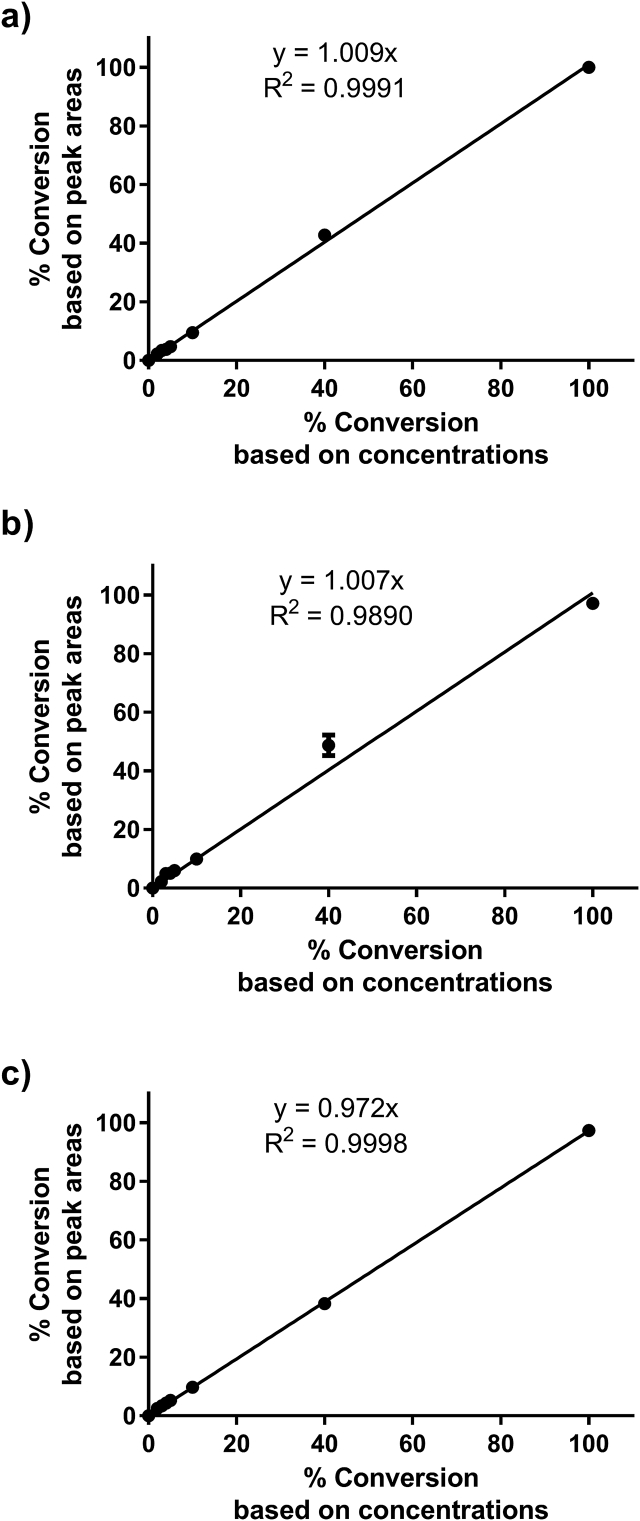
**Calibration curve for GMP conversion into GDP.** Plotted as mean of at least two replicates ± standard deviation (SD). Total summed concentration ([GMP] + [GDP]) of 5 μM in (a), 50 μM in (b) and 100 μM in (c).

The separation achieved by HPLC chromatography allowed unambiguous monitoring of the progress of any guanylate kinase reaction. However, given that the nucleotide standards, when analyzed at the same concentration (50 μM), gave different integrated areas (Fig. 1), a correction factor had to be applied to GDP and ADP areas when calculating percent conversion of substrate into product. While this correction factor was constant regardless of the total summed concentration of GMP and GDP used ([GMP] + [GDP]), the same was not observed for ADP and ATP (potentially due to deviations from the Beer Lambert law at low and high ATP and/or ADP concentrations used). Therefore, only GMP and GDP were used for the calculation of the conversion of substrate into product. Fig. 2 (a, b and c) demonstrates the direct correlation between conversion values obtained by measurement of peak areas using a correction factor of 1.56, and based on actual concentrations of GDP and GMP standards, for total summed concentrations of 5, 50 and 100 μM, respectively (for example, for 20% conversion of substrate into product: 80 μM GMP and 20 μM GDP for total summed concentration of 100 μM; 40 μM GMP and 10 μM GDP for total summed concentration of 50 μM; 4 μM GMP and 1 μM GDP for total summed concentration of 5 μM).

Quality control (QC) samples (n = 5) assessed at 3%, 20% and 100% substrate conversions into product, using total summed concentrations ([GMP] + [GDP]) of 100 μM, demonstrated good intraday accuracy and precision of the method (Table 1). Percent relative standard deviations (%RSD) and percent relative errors (%RE) were all under 15%. To estimate %RE, back calculated concentrations were determined assuming a slope of 1.

**Table 1:** Intraday accuracy and precision of the developed method.

% Conversion based on concentrations	% Conversion based on peak areas of GMP and GDP		
	Mean	%RSD	%RE
QCL (3%)	3.38	0.99	12.75
QCM (20%)	18.54	1.76	−7.31
QCH (100%)	98.01	0.06	−1.99

### 3.2 Optimization of the *Pv*GK enzymatic reaction

**Fig. 3:**
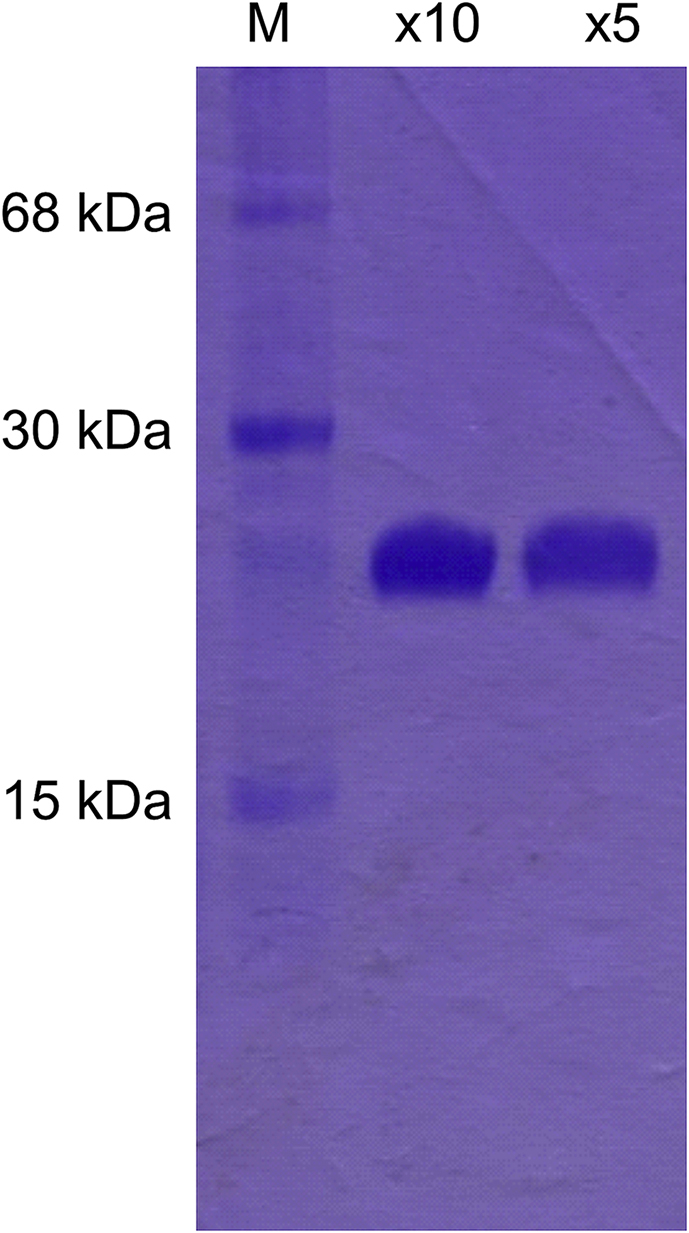
**Coomassie-stained SDS-PAGE of purified *Pv*GK.** Lanes are labelled as follows: M, molecular weight marker; × 10, flow-through from Q-Sepharose column 10-fold concentrated; × 5, flow-through from Q-Sepharose column 5-fold concentrated. The concentration of protein obtained from anion exchange chromatography was 0.2 mg/mL as determined by UV absorption at 280 nm.

Large-scale expression of *Pv*GK was successfully achieved in *E. coli* by auto-induction with 48 h incubation time. The subsequent purification involved just two separation steps: immobilized metal-ion affinity chromatography with a Ni^2+^-NTA column and anion-exchange chromatography with Q-Sepharose. A representative SDS-PAGE is shown in Fig. 3.

To determine the ideal quencher for the enzymatic reaction (50 μL scale), addition of 50 μL of MeOH, 10 μL HCl 32% (v/v), 10 μL EDTA 250 mM and 10 μL HCl 0.1% (v/v), were investigated. Quenching with 10 μL HCl 0.1% (v/v) was found to be the best method. Under these conditions, the pH of the reaction mixture was decreased to 3, which was sufficient to denature the enzyme, but did not interfere with retention time and peak shape of the HPLC separation.

**Fig. 4:**
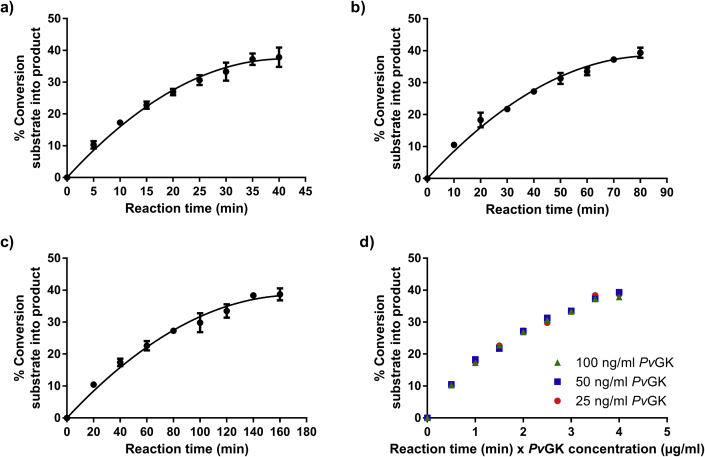
**Reaction progress curves of *Pv*GK catalyzed reaction.** (a) Using 100 ng/mL *Pv*GK over 40 min; (b) using 50 ng/mL *Pv*GK over 80 min; (c) using 25 ng/mL *Pv*GK over 160 min. Plotted as mean of three replicates ± SD; (d) percent conversion of substrate into product plotted against the multiplication between reaction time in min and enzyme concentration in μg/mL.

To determine the best enzyme concentration and incubation time as well as to assess enzyme stability during the course of the assay, three reaction progress experiments were performed on three different days, with three different *Pv*GK concentrations (100, 50 and 25 ng/mL), three corresponding different incubation times (40, 80 and 160 min, respectively) and fixed GMP and ATP substrate concentrations (100 μM). If the enzyme is stable during the course of the enzymatic reaction, for constant concentrations of substrate and any activator or inhibitor, the amount of product formed depends only on the concentration of enzyme and the reaction time [22]. Consequently, the same amount of product should be formed if an enzymatic reaction is performed with half the enzyme concentration and doubled reaction time (Fig. 4). Given that *Pv*GK was stable during the assays, the same curve was obtained independently of the initial concentration of *Pv*GK used (Fig. 4). The combined data did not exceed a %RSD of 6%, demonstrating the good interday precision of the method.

**Fig. 5:**
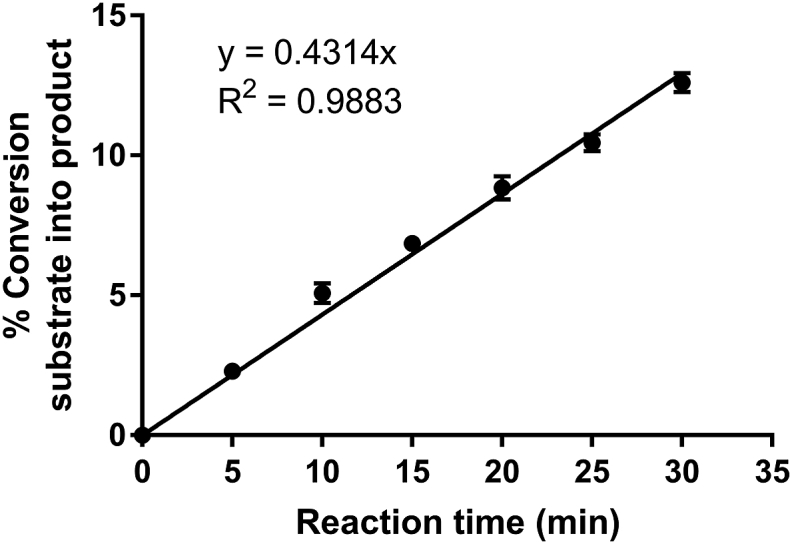
Initial linear rate of *Pv*GK catalyzed reaction. Obtained with 25 ng/mL (1.06 nM) *Pv*GK, 100 μM ATP and GMP and 30 min incubation time. Plotted as mean of three replicates ± SD.

For an enzyme concentration of 25 ng/mL, the initial rate of the reaction (up to 30 min) could be fitted with a linear function (Fig. 5).

### 3.3 Study of *Pv*GK kinetics

The method developed was used to study the kinetics of the forward reaction (forming ADP and GDP) catalyzed by *Pv*GK. Considering that these enzyme kinetic analyses require variation in the initial concentrations of ATP and GMP, incubation times had to be adjusted so that enough data points could be obtained during the linear phase of the progress curves (%Conversion *vs.* time plots) and initial reaction velocities could be appropriately estimated.

**Fig. 6:**
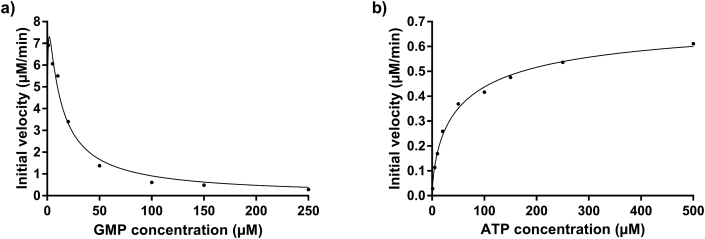
**GMP and ATP saturation curves.** (a) Initial velocities decreased with increasing GMP concentrations, indicating substrate inhibition. (b) ATP saturation curve showing a good fit to a sigmoidal model and negative cooperativity (h = 0.67).

The GMP saturation curve (initial velocity *vs.* concentration of substrate) with fixed ATP concentrations of 500 μM showed a decrease in initial velocity with increasing GMP concentrations, indicating strong substrate inhibition (Fig. 6a). Since GMP concentrations lower than 1 μM could not be tested due to sensitivity limitations of the assay, initial velocities could not be obtained for the upward phase of the saturation curve.

Interestingly, the ATP saturation curve was sigmoidal (Hill coefficient, *h*, of 0.67 ± 0.06) when fixed GMP concentrations of 100 μM were used, indicating negative cooperativity (Fig. 6b). The ATP saturation curves were fitted in GraphPad Prism to the following model: *V*_*0*_ *= V*_*max*_*[S]*^*h*^*/(K*_*prime*_ *+ [S]*^*h*^*)*. An apparent *K*_*prime*_ (a constant related to *K*_*m*_ and used in sigmoidal kinetics) of 15.6 ± 2.07 μM was obtained for ATP. Apparent *V*_*max*_ was estimated as 0.74 ± 0.06 μM/min.

These results show that *Pv*GK enzyme activity is strongly influenced by the relative concentrations between the two substrates GMP and ATP. Substrate inhibition and/or sigmoidal kinetics have been described in yeast guanylate kinase [23], and other regulatory enzymes, such as 3-deoxy-D-arabino-heptulosonate 7-phosphate synthase from *Rhodomicrobium vannielii* [24], ketopantoate reductase from *Staphylococcus aureus* [25], or phosphofructokinase [26].

### *3.4* *Pv*GK *inhibition assay for compound screening and IC*_*50*_*determination*

Twenty fragment-like compounds that emerged from a native mass spectrometry-based binding screening against *Pv*GK were tested [27]. Based on *Pv*GK kinetics and considering sensitivity constraints, the inhibition assays were conducted with 20 μM ATP and 10 μM GMP. All assays were run over a period of three months. Each batch of assays was accompanied by 0% and 100% inhibition controls, the latter obtained by adding 10 μL HCl 0.1% (v/v) before the addition of ATP for reaction start. These controls were pooled together and used to evaluate the assay performance and reproducibility over the course of the experiments. With a total of 81 0% and 100% inhibition controls, a signal window of 3.8 and a Z′ factor of 0.6 were obtained. Since a signal window >2 is generally recommended and a Z’ factor >0.5 is considered very good [28], the suitability of the present assay for compound screening is demonstrated.

**Fig. 7:**
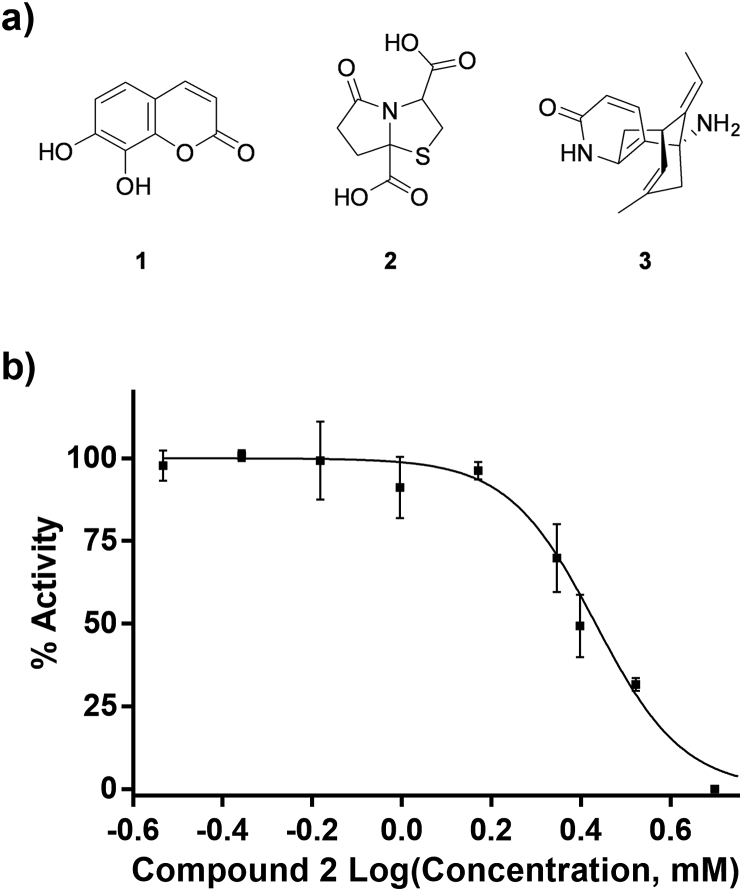
Chemical structures of fragment-like compounds 1, 2 and 3 (a) and half maximal inhibitory concentration (IC_50_) curve for compound 2 (b).

Percent inhibition for each tested fragment was calculated based on 0% and 100% inhibition controls assayed on the same day. Alongside the fragment-like compounds, EDTA and a known broad-spectrum kinase inhibitor with fragment-like physicochemical properties, 7,8-dihydroxycoumarin (daphnetin) (**1,** Fig. 7a), were tested. Percent inhibition in the presence of 5 mM EDTA and daphnetin was 84 and 68%, respectively. EDTA inhibits *Pv*GK activity via a non-specific mechanism involving chelation of Mg^2+^, which is essential for enzymatic activity. In agreement with our results, Kandeel et al. found that EDTA inhibited *P. falciparum* GK by 85% at 3 mM [29]. Daphnetin is a natural product (a coumarin derivative) that has been found to be an inhibitor of epidermal growth factor receptor (EGFR) tyrosine kinase [30] and possess antimalarial activity both *in vitro* and *in vivo* [[31], [32], [33], [34]]. Seven of the tested compounds had percent inhibition higher than 30%. Compound **2** inhibited *Pv*GK activity by 100% at 5 mM and an IC_50_ curve was generated (Fig. 7b). The determined IC_50_ value was 2.7 mM. Compound **3**, known as (−)-Huperzine A, was found to be an activator. (−)-Huperzine A is a reversible acetylcholinesterase inhibitor and has been investigated for the treatment of Alzheimer's disease [35].

## 4 Conclusions

Guanylate kinase activity is often measured using coupled-enzyme systems and/or fluorescent/luminescent probes. Herein, we described the development of an HPLC-based method specific for guanylate kinase activity, which employs direct detection of substrates and products of the enzymatic reaction (GMP, ATP, ADP, GDP) through their intrinsic UV absorption at 260 nm. Chromatographic separation was achieved in 12 min per run with a C18 column operating under isocratic conditions. Within the range of substrate concentrations used, unambiguous determination of the conversion of substrate into product could be achieved using GMP and GDP integrated chromatogram peak areas. Under the experimental conditions described, the method demonstrated good accuracy and precision.

The method was successfully applied to study the activity of *Pv*GK, a potential drug target against malaria. This protein is a soluble, cytosolic regulatory enzyme highly expressed during the liver stage of *Plasmodium* life cycle. Enzyme kinetic analyses revealed that the activity of *Pv*GK depends highly on the relative concentrations of the two substrates GMP and ATP. In addition to enzyme kinetic studies, the present method demonstrated to be suitable for compound screening. Twenty fragment-like compounds were tested in inhibition assays and an IC_50_ curve was generated for the best *Pv*GK inhibitor, compound 2.
